# Association of Hearing Impairment With Higher Level Physical Functioning and Walking Endurance

**DOI:** 10.1093/geroni/igab046.1700

**Published:** 2021-12-17

**Authors:** Pablo Martinez Amezcua, Pei-Lun Kuo, Nicholas Reed, Eleanor Simonsick, Yuri Agrawal, Frank Lin, Jennifer Deal, Jennifer Schrack

**Affiliations:** 1 Columbia University Irving Medical Center, New York, New York, United States; 2 National Institute on Aging, National Institute on Aging, Maryland, United States; 3 Johns Hopkins Bloomberg School of Public Health, Baltimore, Maryland, United States; 4 National Instute on Aging/NIH, Baltimore, Maryland, United States; 5 Otolaryngology, Baltimore, Maryland, United States; 6 Johns Hopkins University, Johns Hopkins University, Maryland, United States; 7 Johns Hopkins University, Baltimore, Maryland, United States

## Abstract

The longitudinal associations between hearing impairment and higher-level functional measures and the potential confounding role of vestibular function have not been assessed. We investigated these associations in 831 participants of the Baltimore Longitudinal Study of Aging (2012–2019). Hearing was measured using pure-tone audiometry and categorized using WHO standards. Physical function was assessed with the Health Aging and Body Composition Physical Performance Battery (HABCPPB, higher=better) and walking endurance with time to walk 400 meters. Multivariable regression models tested the hypotheses that participants with hearing impairment have poorer physical outomes. In a subset, we further adjusted for vestibular function. Hearing impairment was associated with decrements in higher-level physical performance and walking endurance, and faster decline over time, regardless of vestibular function. Among participants with any hearing impairment, hearing aid users were faster in the 400-m walk. Early screening for higher-level functional loss among older adults with hearing loss is warranted.

